# Does excitatory fronto-extracerebral tDCS lead to improved working memory performance?

**DOI:** 10.12688/f1000research.2-219.v2

**Published:** 2013-12-10

**Authors:** Níall Lally, Camilla L. Nord, Vincent Walsh, Jonathan P. Roiser

**Affiliations:** 1 Institute of Cognitive Neuroscience, University College London, London, WC1N 3AR, UK

## Abstract

Evidence suggests that excitatory transcranial direct current stimulation (tDCS) may improve performance on a wide variety of cognitive tasks. Due to the non-invasive and inexpensive nature of the method, harnessing its potential could be particularly useful for the treatment of neuropsychiatric illnesses involving cognitive dysfunction. However, questions remain regarding the efficacious stimulation parameters. Here, using a double-blind between-subjects design, we explored whether 1 mA excitatory (anodal) left dorsolateral prefrontal cortex stimulation with a contralateral extracerebral reference electrode, leads to enhanced working memory performance across two days, relative to sham stimulation. Participants performed the 3-back, a test of working memory, at baseline, and during and immediately following stimulation on two days, separated by 24-48 hours. Active stimulation did not significantly enhance performance versus sham over the course of the experiment. However, exploratory comparisons did reveal a significant effect of stimulation group on performance during the first stimulation phase only, with active stimulation recipients performing better than sham. While these results do not support the hypothesis that dorsolateral prefrontal cortex tDCS boosts working memory, they raise the possibility that its effects may be greatest during early learning stages.

## Introduction

Transcranial direct current stimulation (tDCS) has been utilised as a non-invasive brain stimulation methodology to improve performance on a variety of cognitive tasks in healthy volunteers, including decision-making ^1^, planning ^2^ and working memory ^3, 4^. Due to the minimal risk profile, arising as function of the very low current delivered to the scalp, and the relatively inexpensive nature of the device, it has high potential as a clinical tool. Tentative evidence thus far suggests that tDCS may be effective in ameliorating neurological and psychiatric illnesses associated with cognitive deficits. Prominent amongst these are recent developments in the study of the treatment of addiction, depression, schizophrenia and stroke ^5^. However, questions remain over stimulation condition blinding ^6^, optimal stimulation frequency ^7^ and appropriate electrode placement ^8^ as these parameters can strongly influence the efficacy of the stimulation device, the induced neuronal activity and moreover, the interpretation of stimulation effects on cognitive performance. Questions have also been raised about appropriate behavioural and stimulation controls ^9^.

Many studies have reported enhanced task performance in healthy volunteers following application of the excitatory anodal electrode to a location of the scalp corresponding to a task-relevant region of the brain, including: the temporal parietal junction to enhance social cognition ^10^; inferior frontal cortex to enhance target detection ^11, 12^; Wernicke’s area to enhance visual picture naming ^13^; and dorsolateral prefrontal cortex (DLPFC) to enhance decision making ^1, 14^ and working memory ^3, 4, 7^. The application of the excitatory tDCS electrode to the scalp is thought to cause an increase in neuronal excitability in the stimulated area by altering the resting potential ^15^. To complete the electrical circuit, the reference or inhibitory cathodal electrode must be placed somewhere on the head or body being stimulated.

The majority of studies exploring cognitive enhancement using tDCS have targeted DLPFC as their region of excitation while the inhibitory electrode has typically been placed on the contralateral supra-orbit (or DLPFC). For example, in a single blind investigation using this electrode montage, Ohn *et al.* ^4^ found that 30 minutes of 1 mA tDCS while participants performed the n-back task led to significant improvements in task performance over sham during stimulation only. However, placing the reference electrode on the scalp introduces a potential confound in the interpretation of any resulting behavioural effects: these could arise as a result of excitation, inhibition, or a combination of the two electrodes. The location of the reference electrode, whether extra or intra -cephalic, has been show to play a prominent role in the efficacy of the excitatory electrode ^16^.

The n-back ^17^ is a cognitive task commonly used to assess aspects of executive function, and is thought to engage working memory in particular. Although criticisms have been made with respect to its construct validity ^18^, the n-back has frequently been used in the context of functional neuroimaging experiments in both healthy volunteers and patients with psychiatric and neurological illnesses. The results of these studies consistently implicate a network of brain regions including parietal cortex and DLPFC, which are engaged with increasing cognitive load during n-back performance ^19^. Importantly, altered DLPFC function is associated with several psychiatric conditions ^20– 22^. For example, research using the n-back has identified that the DLPFC is hyperactive in patients diagnosed with major depressive disorder (MDD) ^23, 24^, suggesting that MDD patients may need to use greater resources to achieve the same level of performance. Similar findings have been reported in schizophrenia ^25^.

Working memory dysfunction, and executive function deficits more broadly, have been found across a number of psychopathologies, including attention deficit disorder, autistic spectrum disorder, traumatic brain injury, Alzheimer’s disease, schizophrenia and depression ^26– 28^. Indeed, executive function performance has been identified as a tractable endophenotype to explore across neuropsychiatric illnesses due to its prevalence, particularly in depression and schizophrenia ^29, 30^. Thus, there is significant potential for non-invasive brain stimulation techniques such as tDCS to be applied clinically to ameliorate cognitive dysfunction. However, in order to establish whether tDCS has the potential to improve clinical conditions through modulatory effects on executive function, it is pertinent to first establish the effects of specific stimulation parameters in healthy volunteers.

A recent single-blind within-subjects investigation by Zaehle *et al.* ^31^ demonstrated that 15 minutes of 1 mA excitatory tDCS applied to left DLPFC during rest, with the inhibitory electrode placed ipsilaterally at the mastoid, resulted in enhanced post-stimulation performance on the 2-back task in comparison with cathodal, but not sham, stimulation. Importantly, Zaehle *et al.* ^31^ utilized a fronto-extracephalic montage, which attenuates interpretational difficulties as the reference electrode and its position can affect the efficacy of tDCS and the underlying neuronal activity ^16^; it remains unknown whether contralateral or ipsilateral positioned reference electrodes or bifrontal montages are superior in the stimulation of DLPFC. Furthermore, it remains to be determined whether stimulation during a task or while at rest is more beneficial. Andrews *et al.* ^3^ found tentative evidence to suggest that DLPFC tDCS applied concurrently with a cognitive task may provide more robust effects on subsequent working memory performance that stimulation during rest.

Here, we sought to build on prior research ^3, 4, 31^ by conducting a double-blind between-subjects experiment to examine whether excitatory DLPFC tDCS applied across two days would lead to enhancement of n-back performance during and post-stimulation. Specifically, we assessed whether excitatory fronto-extracerebral DLPFC tDCS, with the reference on the contralateral cheek, could improve performance on the 3-back in healthy volunteers across two stimulation days. We hypothesized that those receiving active stimulation would have greater task performance improvement, relative to baseline, in comparison with sham stimulation recipients.

## Methods

### Participants

Twenty-one (14 females, M = 23.09 years, SD = 3.95) right-handed participants were recruited from the Psychology subject pool at University College London, UK. Participants self-reported no history of mental or neurological illness, current psychiatric medication use, no prior or current participation in another brain stimulation experiment within the previous 24 hours and had normal or corrected to normal vision. There were no significant age ( *t*_(19)_ = 0.211, *P* = 0.835) or gender ( *X ^2^*_(1)_= 1.527, *P* = 0.361) differences between the two stimulation groups (active and sham). All participants provided written informed consent and were compensated for their time. The study was approved by the UCL ethics committee.

**Figure 1.**
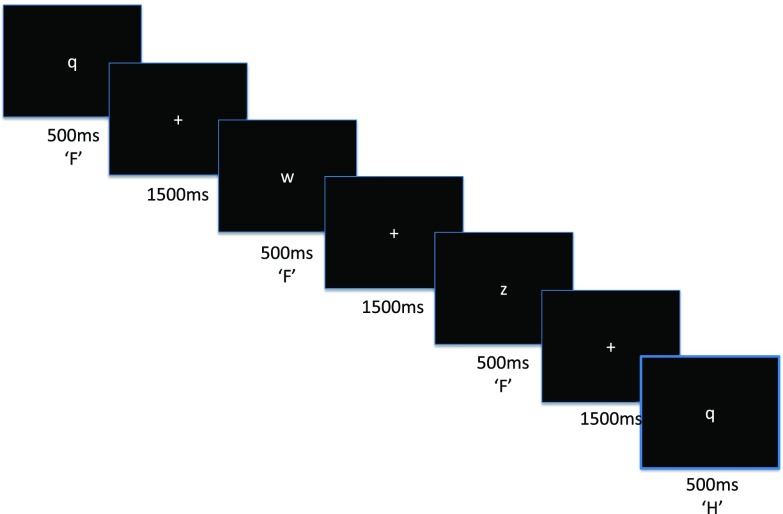
Schema of 3-back task. Stimuli (consonants) were presented centrally for 500 ms and followed by a fixation cross for 1500 ms. Participants were instructed to respond to every stimulus, indicating whether the stimulus matched the letter 3-back (‘H’) or not (‘F’).

***Task***. The n-back ( Figure 1) consisted of a continuous sequence of 300 (150 for baseline) centrally presented consonants (500 ms) interleaved with fixation crosses (1500 ms). Participants were instructed to respond to every appearance of a letter (button pressing did not affect stimulus timing), pressing the ‘H’ key only when the letter onscreen matched the letter 3-back, and pressing the ‘F’ key for all other instances. It is thought that this version of the n-back may afford increased sensitivity to working memory performance than versions that focus solely on hits ^31, 32^. Matches (one-fifth of all stimuli) and non-matches to 3-back stimuli were randomized in order but their ratio was fixed throughout the experiment. The task was coded in Matlab (release 2008b for Windows; Mathworks, Natick, MA, USA) using the Cogent Toolbox ( http://www.vislab.ucl.ac.uk/cogent_2000.php) and is available for free online (available at https://sites.google.com/site/nialllally/home/code/ and https://github.com/nialllally/nback_dprime/blob/master/NBack.zip). The code is also permanently available at 10.5281/zenodo.7148

### tDCS

**Figure 2.**
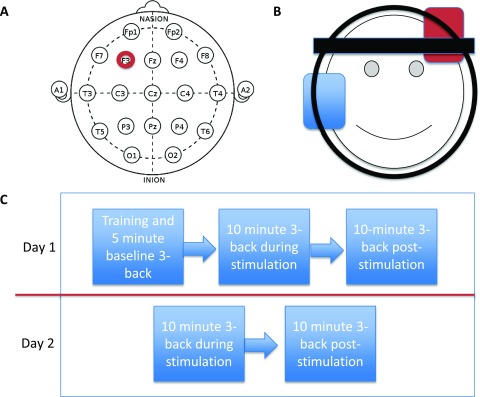
tDCS electrode montage and study design. **A**, **B**) The excitatory anodal electrode (red) was positioned using a 10–20 standard electroencephalography electrode cap under F3, which corresponds to dorsolateral prefrontal cortex. The inhibitory cathodal electrode (blue) was positioned on the contralateral cheek. **C**) Timeline of events in the study. Participants performed the 3-back on five separate occasions, once at baseline on day 1, twice during and twice following stimulation on both days.

tDCS was administered continuously at 1 mA using the Neuroconn DC-Stimulator (Neuroconn, Germany) via a pair of rubber electrodes (7 cm × 5 cm) housed in small synthetic sponges dampened with salt water to increase conductivity. The excitatory (anodal) electrode was placed over F3 ( Figure 2A), corresponding to the left DLPFC, while the reference (cathodal) electrode was placed on the contralateral cheek ^33, 34^. F3 was located using a 10–20 electroencephalography cap and demarcated using a removable marker. Left DLPFC was chosen as the anodal electrode position as this region has been consistently implicated in working memory paradigms ^19^. Additionally as the task involved processing static letters, the left side of the brain was considered most appropriate ^35^. Once the area was located, the electrodes were fastened in position using two headbands (a polyester hairband across the forehead and a rubber band beneath the jaw and around the circumference of the head; Figure 2B).

Before arrival, participants were randomized to one of two brain stimulation conditions using Matlab, active (N=10) or sham (N=11). Specific codes were selected from the tDCS device manual by an independent researcher not involved with the study and were assigned to each condition and randomized to each participant. Importantly, utilizing the ‘study mode’ of the device allowed the stimulation-administering researchers to remain blinded to the condition participants were in as the readout on the stimulation apparatus was identical for both active and sham stimulation. However, the integrity of the blinding was not assessed. The administered current was applied for 10 minutes with an additional 15-second fade-in and fade-out ramping period to minimize discomfort and facilitate participant blinding. Sham stimulation was limited to small pulses of 100–200 µA every 400–550 ms between a 15-second fade-in and fade-out voltage ramp ^36^.

### Study design

During the baseline session on day 1 (D1), participants were first trained on the n-back with a brief exposure to 1, 2 and finally 3-back. Thereafter, participants completed a 5-minute version of the 3-back, which served as a baseline pre-stimulation measure of performance. Immediately after, tDCS was administered for 10 minutes while participants performed the 3-back task (D1 tDCS; Figure 2C). Following this, participants completed a further 10-minute session of the 3-back (D1 post-tDCS) without stimulation. Participants were instructed that continuation to day 2 was dependent on D1 post-tDCS task performance but were not given feedback until the end of day 1. Continuation to day 2 was dependent on above chance performance on the D1 post-tDCS assessment only, which was any positive d' value:

                                                       d' = Z(hit rate) - Z(false alarm rate)                 (1)

where hit and false alarm rate are the number of correct or incorrect ‘H’ responses, respectively, divided by the total number of opportunities (1/5 or 4/5 of total stimulus letters) and Z is the inverse of the cumulative Gaussian distribution. Participants received £10 for their participation on day 1 irrespective of task performance. Day 2 (24–48 hours later) consisted of one 10-minute task run with stimulation (D2 tDCS) and one post-stimulation (D2 post-tDCS). Participants were told that performing better than the test phase of day 1 would result in a bonus of £10 on top of the £5 basic payment on day 2. Thus, participants had a performance incentive for the post-tDCS assessment only on each day.

### Statistical analyses

To assess the effect of active stimulation versus sham over time, we conducted a linear mixed model in SPSS, version 21 (IBM Corp New York 2012). The dependent variable was d'. Follow up models also were conducted using hit rate, correct rejection rate (1 - false alarm rate) and reaction time for both of these variables. The four testing sessions after baseline (D1 tDCS, D1 post-tDCS, D2 tDCS, D2 post-tDCS) were entered as a fixed effect of time; tDCS and sham stimulation were entered as a fixed effect of group; and their interaction (time-by-group) was also entered as a fixed effect. Participant number was entered as a random effect and baseline performance was entered as a covariate. A heterogeneous first order autoregressive covariance structure was employed. Bonferonni corrected tests between the groups at each time point were conducted using linear contrasts to assess between-group differences. Follow up assessments of significant points were assessed using a general linear model with baseline performance entered as a covariate. Performance differences at baseline were assessed using an independent sample t-test. Based on our sample size we had 80% power to detect a large effect size (d=1.3) at *P* = 0.05 (two-tailed) between the stimulation groups.

## Results

**Figure 3.**
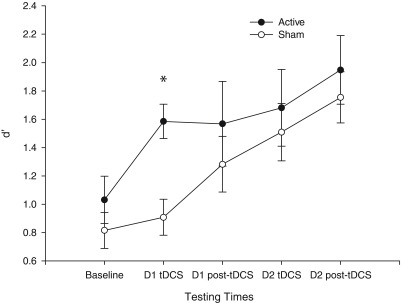
3-back d' performance (mean values) across testing times and days. The active stimulation group always performed better than the sham group but only statistically significantly so during stimulation on day 1 (D1 tDCS), denoted by an asterisk (*). Baseline performance did not differ between the groups but was included in the model as a covariate. Error bars represent ±1 standard error of the mean.

One participant (sham group) scored a negative d' value for day 1 and did not participate in session 2, but their data were included in the linear mixed model. Additionally, the testing computer malfunctioned during the day 1 post-tDCS assessment for 1 participant (active group), approximately 40% through the task; these data were included in the model and the participant completed a further post-tDCS test, which was used only to determine progress to day 2. There was no significant difference in d' performance between the groups at baseline ( *t*_(19)_ = 1.044, *P* = 0.309; Figure 3). As expected, there was a significant main effect of time ( *F*_(3,36)_ = 7.669, *P* < 0.001) on d' performance, reflecting improvement across both groups with increasing exposure to the task. However, contrary to our hypothesis, no main effect of stimulation group was identified ( *F*_(1,16)_ = 2.228, *P* = 0.155) and there was no group × time interaction ( *F*_(3,36)_ = 1.339, *P* = 0.277).

Exploratory Bonferonni corrected pairwise comparisons were carried out to assess group performance differences at the four post-baseline time points. A significant difference between active sham stimulation was identified at the day 1 tDCS time point ( *F*_(1,13.373)_ = 10.747, *P* = 0.006; controlling for baseline performance and Bonferonni corrected for multiple comparisons), indicating a large effect size (Cohen’s *d* = 1.427, *r* ^2^ = 0.337). No other stimulation group differences in d' were found at other time points (all *F* < 1.2, *P* *>* 0.3; see Table 1 for group performance across sessions and task components). Further analyses of performance during stimulation on day 1 (including baseline as a covariate) revealed that both the hit rate ( *F*_(1,18)_ = 4.454, *P* = 0.049, ηp ^2^ = 0.198) and correct rejection rate ( *F*_(1,18)_ = 3.680, *P* = 0.071, ηp ^2^ = .170) of active stimulation recipients were significantly, or at trend level, better than sham. However, no significant reaction time differences were found for this time point for either hits ( *F*_(1,18)_ = 0.010, *P* = 0.923, ηp ^2^ = 0.001) or correct rejections ( *F*_(1,18)_ = 0.202, *P* = 0.659, ηp ^2^ = 0.011).

**Table 1.** Means and standard deviations of each 3-back session per group. D1 = day 1, HR = hit rate, CRR = correct rejection rate, RT = reaction time, CR = correct rejection.

		Baseline	D1 tDCS	D1 post-tDCS	D2 tDCS	D2 post-tDCS
**Anodal**	HR	0.4167 (0.1694)	0.5250 (0.1336)	0.5333 (0.2278)	0.5533 (0.2131)	0.6067 (0.1994)
	CRR	0.8933 (0.0355)	0.9296 (0.0385)	0.9317 (0.0486)	0.9283 (0.0500)	0.9425 (0.0389)
	Hit RT	0.6848 (0.2587)	0.6651 (0.2358)	0.6300 (0.2467)	0.6052 (0.2279)	0.6118 (0.2609)
	CR RT	0.6702 (0.2422)	0.6566 (0.2159)	0.6180 (0.2214)	0.5948 (0.2229)	0.5939 (0.2237)
**Sham**	HR	0.3909 (0.1106)	0.4030 (0.1197)	0.4879 (0.1959)	0.5030 (0.2048)	0.5333 (0.2196)
	CRR	0.8614 (0.0429)	0.8720 (0.0577)	0.9049 (0.0383)	0.8186 (0.2757)	0.8390 (0.2821)
	Hit RT	0.7026 (0.2062)	0.6816 (0.1763)	0.6345 (0.1770)	0.5546 (0.2339)	0.5714 (0.2734)
	CR RT	0.7159 (0.2017)	0.6780 (0.1715)	0.6365 (0.1630)	0.5727 (0.2448)	0.5592 (0.2685)

3-back scores of participants before, during and after tDCS stimulation to their left dorsolateral prefronatal cortex Files are named by date, subject code, day and task condition. There are four different conditions: Exposure, Baseline, DCS and Test. Exposure was a brief training on the n-back task. Baseline was a 5 minute period of the 3-back. Exposure and Baseline occurred only on day 1. DCS stands for direct current stimulation and indicates performance of the 3-back (10 minutes) that occurred while participants were stimulated. The test condition (10 minutes of 3-back) occurred post-stimulation. Both DCS and the Test conditions occurred on days 1 and 2.
For example: 01-Jan-2012_AA01_1_DCS - indicates the performance during stimulation on day 1 01-Jan-2012_AA01_2_Test - indicates the performance during post-stimulation test phase on day 2
Variables in the files are presented in the following order:
shudaprsd = indicates instances where the letter on screen was the same as the letter 3-back (1) or not (0) key_press = the key pressed (6 = F, letter on screen is not the same as 3-back; 8 = h, letter on screen is the same as letter 3-back) instructions = time relative to the start of the file when the instructions appeared on screen fixation = time relative to the start of the file when the first fixation cross appeared on screen letter_onscreen = time relative to the start of the file when the letter appeared on screen stimulus_interval = time relative to the start of the file when the fixation cross disappeared off screen RT = reaction time in seconds from the time stimulus onset to the button press Hits = the amount of correct hits (3-back) CRs = the number of correct rejections FAs = false alarms, pressing the hit button at the wrong occasion Misses = incorrect rejection of hit trial (opposite to false alarm) or = omitted rejection response oh = omitted hit response HR = hit rate calculated as the number of hits per chances FAR = false alarm rate calculated as the number of false alarms per opportunity Z_HR = Z normalised hit rate, where Z is the inverse of the cumulative Gaussian distribution Z_FAR = Z normalised false alarm rate, where Z is the inverse of the cumulative Gaussian distribution d = d prime calculated as Z_HR - Z_FAR

## Discussion

Deficits in executive function, including working memory, have been implicated in many neurological and psychiatric conditions and have also been targeted as potentially tractable endophenotypes in both schizophrenia ^37^ and depression ^29^. Although tDCS has been suggested as a therapeutic tool for many cognitive and neurological impairments, very few tDCS studies have conducted double-blind assessments in either clinical or non-clinical populations. Furthermore, the specific ameliorative stimulation parameters, such as amplitude, frequency and electrode positioning, are largely undefined. Importantly, few electrode montages have been tested ^1, 31, 38, 39^, fewer studies yet have applied tDCS for greater than one session ^40– 42^ and many of the variables in the available parameter space have not been subject to systematic manipulation ^9^. Contrary to our hypothesis, no effect of tDCS on task performance was identified in this study. However, exploratory tests did suggest that active stimulation was associated with enhanced performance relative to sham stimulation during the first stimulation period on day 1 only. Our results may therefore indicate that the performance enhancement effects of excitatory tDCS may be limited to earlier stages of learning ^42^. They also suggest that reports of improvements after one session of tDCS – the most common report in enhancement studies – may not translate to continual improvement with additional stimulation. Indeed, Martin *et al*., ^43^ using an extracephalic electrode montage with the reference placed on the deltoid muscle, found in a relatively large university sample that, in comparison to sham stimulation, repeated sessions (10) of DLPFC tDCS did not lead to a significant improvement in task performance on a variant of the n-back task when controlling for baseline differences. However, uncorrected contrasts, without controlling for baseline, did reveal a benefit in comparison to sham at the first and eight tDCS sessions only; a result largely consistent with our findings here of attenuated effects of tDCS on task performance with repeated sessions.

Our results do not support the hypothesis that excitatory tDCS applied to DLPFC results in post-stimulation improvement on the n-back task across multiple days. This result is consistent with some previous research; in comparison with sham stimulation, neither Zaehle *et al.* ^31^ nor Ohn *et al.* ^4^ demonstrated significant performance enhancements on the n-back task immediately following excitatory DLPFC tDCS. Nevertheless, we did find evidence for a specific improvement in performance during stimulation on day 1 only, an outcome consistent with results from Ohn *et al.* ^4^ and others ^44^. Andrews *et al.* ^3^ found that DLPFC excitatory tDCS applied during a working memory task (n-back) led to significant improvements in post-stimulation performance in comparison with baseline on an alternative working memory task (digit span forward but not backward). The improvements found ^3^ were not present for either sham stimulation in conjunction with task performance or stimulation without task performance. Behavioural data were not reported for the task during stimulation and an intracerebral reference electrode was used, limiting direct comparison with the present study. Furthermore, Hoy *et al.* ^7^ found that 1 mA excitatory tDCS applied to DLPFC at rest resulted in an enhancement in 2-back reaction times 40 minutes post-stimulation, but found no improvement in accuracy. However, other reports have found evidence for more enduring cognitive enhancement following tDCS ^2, 41^ (but see Walsh ^9^).

This discrepancy between results may reflect the different tasks, stimulation parameters, sample sizes and study designs used. For example, it is possible that the payment schemes that served as a performance motivator here limited the potential to observe performance enhancing effects of tDCS. As there was no monetary motivation during the stimulation phase on day 1, participants may not have exerted themselves fully and thus the effects of stimulation may have had greater leverage; while on day 2, participants in both groups may have reached a level whereby any potential for further enhancement of performance through tDCS was limited. Whilst the sample size used here is low for a between-subjects study, few tDCS studies have thus far been conducted using large sample sizes, and future studies should address the issue of stimulation parameter optimization using large sample sizes. Nevertheless, our results suggest that tDCS may be particularly sensitive to earlier stages of learning ^42^.

In theory, the beneficial effects of tDCS may be most pronounced in poorer performers. Indeed, there is some evidence that tDCS may be particularly useful as a cognitive enhancer with lower performing individuals ^34^. As the population utilised here primarily comprised students from University College London, between-group differences arising as a function of tDCS may have been attenuated due to high initial baseline ability. Finally, evidence suggests that individual differences in genotype may play a large part in susceptibility to the plasticity enhancing capabilities of tDCS. Fritsch *et al.* ^45^ found that tDCS was more efficacious in both mice and humans possessing the homozygous Val/Val genotype of the brain-derived neurotrophic factor polymorphism (rs6265), than Met carriers, though we did not have a sufficiently large sample to explore such moderators in the current study.

The electrode montage used here (fronto-extracerebral) may have also played a significant part in the efficacy of the stimulation. While DLPFC is one of the most frequent site selections for anodal tDCS, placing the reference (cathodal) electrode on the contralateral cheek is a relatively novel occurrence ^33, 34^. Current modeling, a technique to determine the amount of electrical current cerebrally induced as a function of the electrode positioning and other parameters, has been performed for various anodal DLPFC montages, with the reference electrode placed on the contralateral supraorbit, DLPFC and deltoid. The DLPFC and contralateral cheek montage however has yet to be modeled, thus it is unknown if it is more or less efficacious than other electrode configurations. Nevertheless, modeling studies have determined that the distance between the anodal and reference electrode is negatively correlated with the current induction magnitude ^16^; in theory, the cheek may be preferential to the deltoid, however this remains to be assessed. Furthermore, the majority of early investigations of tDCS for task performance enhancement used an electrode montage with both electrodes positioned proximal to the cerebrum (e.g. DLPFC and the contralateral supraorbit), which limits the interpretation as to which brain region was primarily modulated as both electrodes could contribute, and may in fact do so in opposing ways, to alterations in task performance. Nevertheless, widespread changes in neuronal activity outside of the sites of stimulation have been consistently identified ^46, 47^, making the issue of electrode placement complex. In sum, electrode positioning is a major potential confound when comparing tDCS effects on both brain activity and behavior across studies, further research addressing the biological and behavioral consequences of differing electrode placement is needed.

The results of this experiment require careful replication and extension to validate the potential role for tDCS in executive function enhancement. In particular, the evaluation of result specificity represents a prominent hole in the current literature. Few studies thus far have contrasted active stimulation results in comparison with control tasks and active stimulation of control site locations on the scalp ^9^; such measures would be beneficial in assessing the findings here and across the field. Additionally, it could be fruitful to replicate this experiment without the monetary incentive. Testing a larger and more representative sample including non-university students would also be informative. Furthermore, while performance improvements under stimulation are important, the clinical utilization of tDCS necessitates long lasting effects once stimulation has ceased. As many psychiatric and neurological illnesses are associated with deficits in executive function task performance, inclusion of a patient group may permit the assessment of the viability of tDCS as a neuroenhancement methodology for psychiatric illnesses. Recent research has indeed shown that tDCS can enhance cognitive control, a component of executive function, in MDD ^48^; however, long-lasting cognitive ameliorative effects of stimulation in depression have yet to be demonstrated.

In conclusion, our results do not support the hypothesis that excitatory tDCS applied to the left DLPFC using a contralateral fronto-extracerebral electrode montage produces consistent improvements in executive function beyond the period of stimulation. Nonetheless, we found a beneficial effect of tDCS during task performance only when the task was relatively novel, which could be interpreted as indicating that this particular electrode montage, stimulation voltage and study design may be best suited to early stages of learning.
