# Supplementary material for: Does excitatory fronto-extracerebral tDCS lead to improved working memory performance?
Source: F1000Res. 2013 Dec 10;2:219. Originally published 2013 Oct 17. [Version 2] doi: 10.12688/f1000research.2-219.v2 (PMC3869492; doi:10.12688/f1000research.2-219.v2)
Supplement: Peer review history

# Peer review: Referee response for version 2

**DOI:** 10.5256/f1000research.3252.r2716

**Published:** 2014-03-18

**Authors:** Marian Berryhill

**Affiliations:** 1 Department of Psychology, University of Nevada, Reno, NV, USA

I believe the authors have made the appropriate revisions.

I have read this submission. I believe that I have an appropriate level of expertise to confirm that it is of an acceptable scientific standard.

# Peer review: Referee response for version 2

**DOI:** 10.5256/f1000research.3252.r2717

**Published:** 2014-02-13

**Authors:** Angelo Alonzo

**Affiliations:** 1 School of Psychiatry, University of New South Wales, Sydney, Australia

I thank the authors for the revised article and in particular addressing in detail the electrode placement issue. I agree that interpretation of results from tDCS studies may need to be qualified when using two cerebral electrodes, and that using an extracerebral or extracephalic reference electrode can in theory avoid this ambiguity. However, it should not be assumed that the reduced cortical excitability that can result when using such montages due to the greater interelectrode distances necessarily results in reduced efficacy in therapeutic/behavioural applications of tDCS. This is due to a conceivably greater cerebral area being modulated when for example using an extracephalic reference, which when treating major depressive disorder, may be just as effective (if not more) than a 'bi-cerebral' montage, as it may activate a larger network of brain regions involved in depression. As the authors note, widespread stimulation outside the electrode sites is a characteristic of tDCS and assumptions that effects are limited to the electrode sites are overly simplistic.

I have read this submission. I believe that I have an appropriate level of expertise to confirm that it is of an acceptable scientific standard.

# Peer review: Referee response for version 2

**DOI:** 10.5256/f1000research.3252.r2715

**Published:** 2013-12-18

**Authors:** Andrea Antal

**Affiliations:** 1 Department of Clinical Neurophysiology, Göttingen University, Göttingen, Germany

Thank you for the clarifications, I have no further questions.

I have read this submission. I believe that I have an appropriate level of expertise to confirm that it is of an acceptable scientific standard.

# Peer review: Referee response for version 1

**DOI:** 10.5256/f1000research.2634.r2398

**Published:** 2013-11-22

**Authors:** Angelo Alonzo

**Affiliations:** 1 School of Psychiatry, University of New South Wales, Sydney, Australia

This study investigates a topic of rapidly growing interest in the field of non-invasive brain stimulation techniques – namely using these techniques to improve cognitive abilities.

This article is well referenced although I refer the authors to a recent article by Martin *et al.* ^1^ which found enhancing effects of tDCS on a dual n-back task over 10 successive sessions. Although the present study found no clear difference between active and sham conditions except during tDCS on Day 1, a clearer advantage of tDCS may nonetheless have been observed with additional sessions.

The statistical analyses conducted have been well detailed and I believe are appropriate for the questions of interest. The limitations of the study have also been adequately discussed. The authors rightly point out that the way in which the monetary incentive for participation was managed could have differentially affected performance between the two testing days, thus possibly obscuring any enhancing effects of tDCS. As also noted, the fact that the sample was primarily comprised of university students may have lessened the likelihood of observing tDCS effects, although Martin *et al*. ^1^ similarly recruited a university student sample. Perhaps utilising a more difficult task, for example a dual n-back task or adjusting the n value based on participant performance on the previous trial block, may have produced greater differences in performance and allowed any potential effects of tDCS to be borne out.

Regarding other methodological aspects, the authors applied quite an unusual placement for the reference electrode on the contralateral cheek; one that I have never encountered before. This would not be considered an extracephalic montage as conventionally, an extracephalic electrode is one that has been placed off the head (e.g., deltoid). I am curious as to why the authors chose this montage and if there is any modelling data to support this. Also, were participants asked to guess their stimulation condition after each session? I note that a double-blind design was used but the success of blinding should still be tested.

In short, I commend the authors in investigating the potential uses of tDCS as a clinical tool but given the above limitations, changes in methodology would need to be implemented before drawing firmer conclusions.

I have read this submission. I believe that I have an appropriate level of expertise to confirm that it is of an acceptable scientific standard, however I have significant reservations, as outlined above.

## References

[1] MartinDMLiuRAlonzoA: Can transcranial direct current stimulation enhance outcomes from cognitive training? A randomized controlled trial in healthy participants.*Int J Neuropsychopharmacol*.2013;16(9) : 2191927-193610.1017/S146114571300053923719048

# Response

**Published:** 2013-12-10

**Authors:** Niall Lally

**Affiliations:** University College London, Ireland

We thank Dr. Alonzo for the detailed review, kind comments and suggestions. We have altered the title of the article to better reflect the electrode locations used here. Furthermore, we have attempted to address concerns surrounding the electrode montage by adding references to articles that have used the contralateral cheek as a reference, and added a paragraph to the discussion section detailing the issue of the reference electrode in tDCS experiments. As astutely indicated by Dr. Alonzo, the montage used is somewhat novel and current modelling studies comparing this configuration to other more established montages have yet to be performed. Finally, we thank Dr. Alonzo for the suggested article ( Martin *et al*., 2013).

# Peer review: Referee response for version 1

**DOI:** 10.5256/f1000research.2634.r2527

**Published:** 2013-11-21

**Authors:** Andrea Antal

**Affiliations:** 1 Department of Clinical Neurophysiology, Göttingen University, Göttingen, Germany

**Introduction: **The authors cite previous studies (e.g., Zaehle *et al*., 2011) as an example that the applied electrode montage makes the interpretation of the effect of previous tDCS results difficult, due to the confounding factor coming from the cephalic or extracephalic position of the return electrode. However, the present study addresses this question relatively shortly, limiting the question to the extracephalic reference electrode position. Please rewrite this sentence.

**Method: **Why did you include 21 subjects? Did the task last for exactly the same time for each participant (irrespectively of the individual differences in the reaction time)? Why did the participants receive performance-dependent extra monetary reward on Day 2 only? It makes the comparison between Day 2 and the previous session rather difficult.

**Results: **First paragraph: line 1-5 and line 6 is seemingly contradictory.

**Discussion: **page 5: ” *no effect of tDCS* “ delete stimulation, and: “ *was identified*” on what?

I have read this submission. I believe that I have an appropriate level of expertise to confirm that it is of an acceptable scientific standard.

# Response

**Published:** 2013-12-10

**Authors:** Niall Lally

**Affiliations:** University College London, Ireland

We thank Dr. Antal for the comments and suggestions. We have amended the manuscript to address the majority of comments.

# Peer review: Referee response for version 1

**DOI:** 10.5256/f1000research.2634.r2142

**Published:** 2013-11-15

**Authors:** Marian Berryhill

**Affiliations:** 1 Department of Psychology, University of Nevada, Reno, NV, USA

In this article, the authors provide a between-subjects comparison of 3-back performances after active and anodal tDCS to the left DLPFC. The first question of interest was to identify the time course of potential tDCS performance modulations and the second, to determine whether extracephalic (contralateral cheek) influenced performance. A significant benefit of active tDCS was identified immediately after the first session, but was not seen not during the first or second sessions. 

The experiment appears to be very well done and the writing clearly explains their process. My primary concern is that the authors conducted this experiment using a very small sample size (which they acknowledge, and provide power estimates that are quite reasonable). However, tDCS effects in healthy young university students tend to be quite modest. Ideally, the authors would replicate the experiment with a larger sample and/or with a secondary WM task (apart from the baseline covariate) to identify any heterogeneities in the population that could be obscuring equal and opposite effects. More importantly, to provide greater generalizability in their data, it would be good for them to compare the cheek site with the commonly used orbital site. This would allow greater comparison between the current study and the existing research, and strengthen their point that different paradigms may elicit different effects.

My second point is that the authors do not really return to the issue of the second electrode site as a potential explanation for their modest effects. One possible explanation would be that the previous work stimulating the contralateral supraorbital region provided stimulation elsewhere in the PFC which contributed to the WM benefit reported by others. Here, current modelling would be particularly helpful to compare the flow of current between the L. DLPFC-R. cheek vs. L-DLPFC-R.supraorbital montages. However, it is clear that the full parameter-space of tDCS effects requires increased clarity and a forum to avoid the 'file-drawer problem' associated with neurostimulation and small effect sizes. Overall therefore, these data will be of interest to the tDCS research community.

I have read this submission. I believe that I have an appropriate level of expertise to confirm that it is of an acceptable scientific standard, however I have significant reservations, as outlined above.

# Response

**Published:** 2013-12-10

**Authors:** Niall Lally

**Affiliations:** University College London, Ireland

We thank Dr. Berryhill for the helpful comments. We agree that the issue of the return electrode is critical in the tDCS literature and remains to be fully evaluated. Therefore, we have revised the manuscript to include a discussion of the issue of the reference electrode in this experiment and identified issues which need to be addressed in future experiments.
